# Crystal structures and hydrogen bonding in the morpholinium salts of four phen­oxy­acetic acid analogues

**DOI:** 10.1107/S2056989015019842

**Published:** 2015-10-28

**Authors:** Graham Smith, Daniel E. Lynch

**Affiliations:** a Science and Engineering Faculty, Queensland University of Technology, GPO Box 2434, Brisbane, Queensland 4001, Australia; b Exilica Ltd., The Technocentre, Puma Way, Coventry CV1 2TT, England

**Keywords:** crystal structure, morpholine salts, phen­oxy­acetic acids, herbicides, 2,4-D, 3,5-D, hydrogen bonding

## Abstract

The anhydrous salts morpholinium (tetra­hydro-2-*H*-1,4-oxazin-4-ium) phen­oxy­acetate, C_4_H_10_NO^+^·C_8_H_7_O_3_^−^, (I), morpholinium (4-fluoro­phen­oxy)acetate, C_4_H_10_NO^+^·C_8_H_6 _FO_3_^−^, (II), and isomeric morpholinium (3,5-di­chloro­phen­oxy)acetate (3,5-D), (III), and morpholinium (2,4-di­chloro­phen­oxy)acetic acid (2,4-D), C_4_H_10_NO^+^·C_8_H_5_Cl_2_O_3_^−^, (IV), have been determined and their hydrogen-bonded structures are described. In the crystals of (I), (III) and (IV), one of the the aminium H atoms is involved in a three-centre asymmetric cation–anion N—H⋯O,O′ *R*_1_^2^(4) hydrogen-bonding inter­action with the two carboxyl O-atom acceptors of the anion. With the structure of (II), the primary N—H⋯O inter­action is linear. In the structures of (I), (II) and (III), the second N—H⋯O_carbox­yl_ hydrogen bond generates one-dimensional chain structures extending in all cases along [100]. With (IV), the ion pairs are linked though inversion-related N—H⋯O hydrogen bonds [graph set *R*_4_^2^(8)], giving a cyclic hetero­tetra­meric structure.

## Abstract 2

The anhydrous morpholinium salts of phen­oxy­acetic acid, (4-fluoro­phen­oxy)acetic acid and the isomeric (3,5-di­chloro­phen­oxy)acetic acid and (2,4-di­chloro­phen­oxy)acetic acid, provide three similar examples of one-dimensional hydrogen-bonded chain polymers and one of a cyclic hydrogen-bonded hetero­tetra­mer.

## Chemical context   

Morpholine (tetra­hydro-2-*H*-1,4-oxazine) is an moderately strong base (p*K_a_* = 8.33) and forms salts with a number of organic acids, some having medical applications, *e.g.* the salicylate (retarcyl, depasol), used as an analgesic, an anti­pyretic and an anti-inflammatory agent (O’Neil, 2001 ▸). The crystal structures of a number of these morpholinate compounds have been reported, some examples of salts with substituted benzoic acids being the 4-amino­salicylate (André *et al.*, 2009 ▸), and a series of isomeric chloro­nitro­benzoates (2,4-, 2,5-, 4,2-, 4,3- and 5,2-) (Ishida *et al.*, 2001*a* ▸,*b* ▸,*c* ▸). In these, cation–anion hydrogen-bonding inter­actions generate either one-dimensional chains or discrete cyclic hetero­tetra­meric structures. Of inter­est is the mode of hydrogen bonding in crystals of the morpholinium salts of some phen­oxy­acetic acid analogues, no structures of which have been reported previously. The reaction of morpholine with phen­oxy­acetic acid (PAA), (4-fluoro­phen­oxy)acetic acid (PFPA) and with the two isomeric homologues, (3,5-di­chloro­phen­oxy)acetic acid (3,5-D) and the herbicidally active (2,4-di­chloro­phen­oxy)acetic acid (2,4-D) (Zumdahl, 2010 ▸), gave the anhydrous salts (I)–(IV), respectively. Their structures and hydrogen-bonding modes are reported on herein.
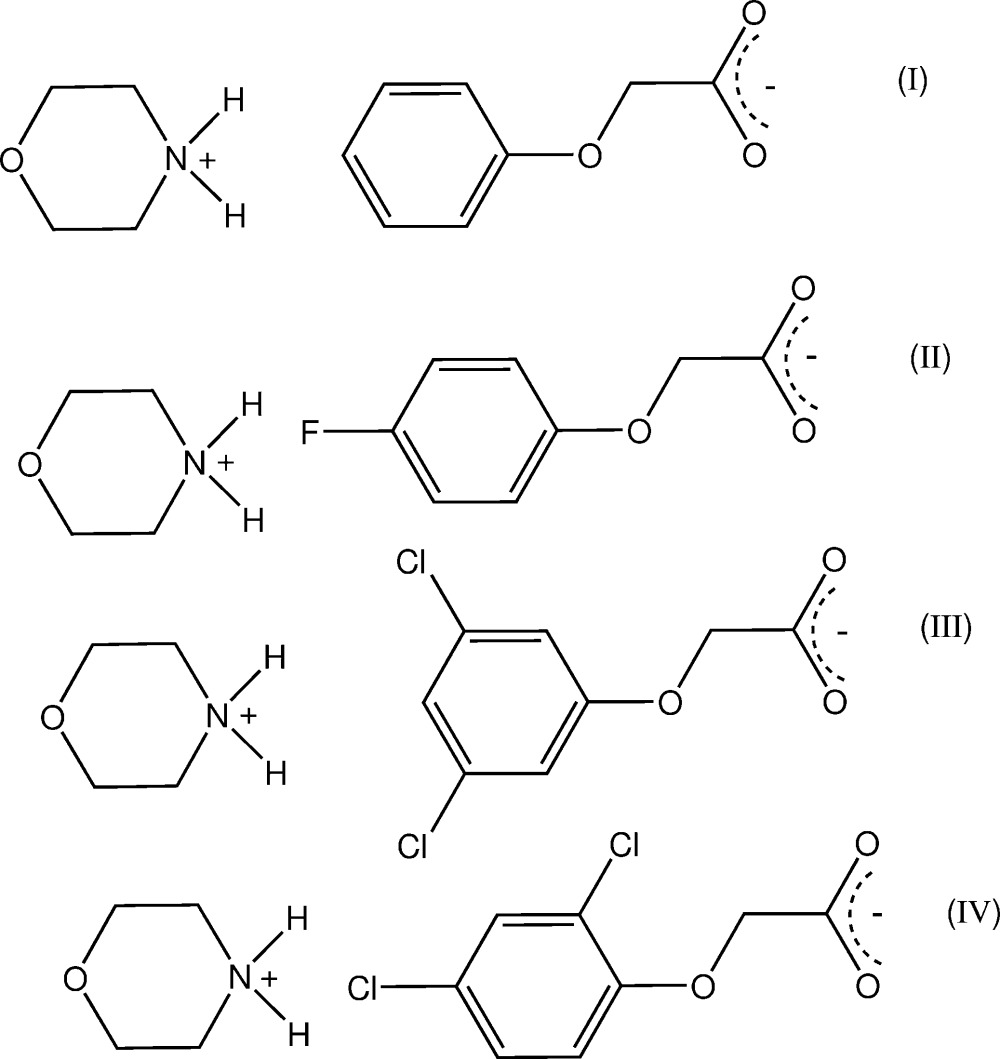


## Structural commentary   

**Figure 1:**
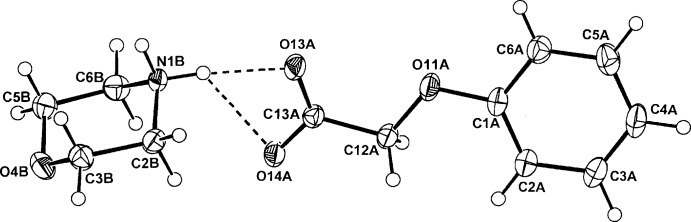
The atom-numbering scheme and the mol­ecular conformation of the morpholinium cation (*B*) and the phen­oxy­acetate anion (*A*) in (I), with displacement ellipsoids drawn at the 40% probability level. The cation–anion hydrogen bonds are shown as dashed lines.

**Figure 2:**
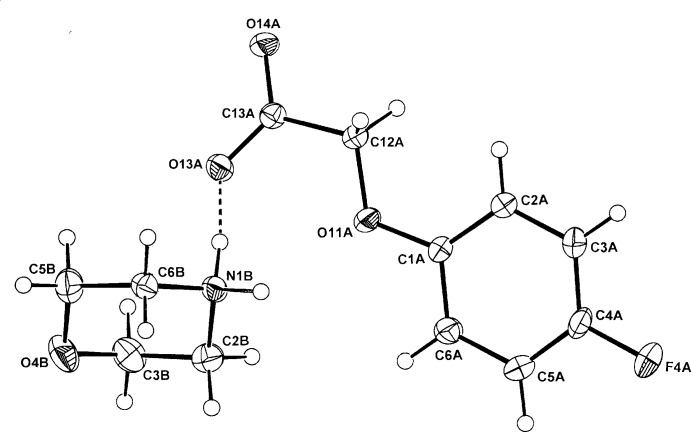
The atom-numbering scheme and the mol­ecular conformation of the morpholinium cation (*B*) and the 4-fluoro­phen­oxy)acetate anion (*A*) in (II), with displacement ellipsoids drawn at the 40% probability level. The cation–anion hydrogen bond is shown as a dashed line.

**Figure 3:**
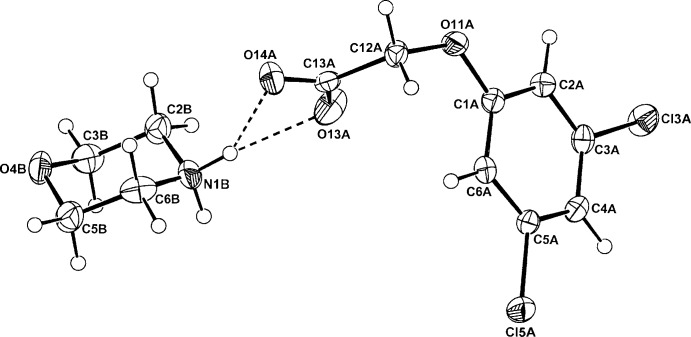
The atom-numbering scheme and the mol­ecular conformation of the morpholinium cation (*B*) and the 3,5-D anion (*A*) in (III), with displacement ellipsoids drawn at the 40% probability level. The cation–anion hydrogen bonds are shown as dashed lines.

**Figure 4:**
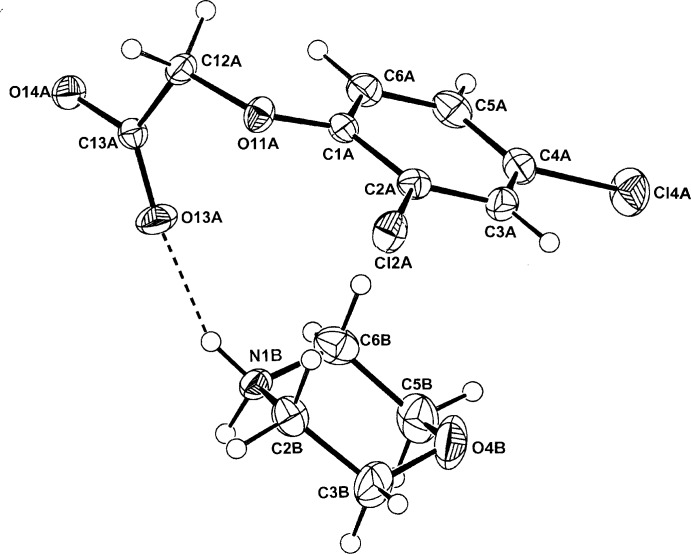
The atom-numbering scheme and the mol­ecular conformation of the morpholinium cation (*B*) and the 2,4-D anion (*A*) in (IV), with displacement ellipsoids drawn at the 40% probability level. The cation–anion hydrogen bonds are shown as dashed lines.

The asymmetric units of (I)–(IV) comprise a morpholinium cation (*B*) and a phen­oxy­acetate anion (*A*) in (I) (Fig. 1 ▸), a (4-fluoro­phen­oxy)acetate anion (*A*) in (II) (Fig. 2 ▸), a 3,5-di­chloro­phen­oxy­acetate anion (*A*) in (III) (Fig. 3 ▸) and a (2,4-di­chloro­phen­oxy)acetate anion (*A*) in (IV) (Fig. 4 ▸). The conformation of the oxo­acetate side chains in the anions of (I) and (II) are essentially planar, with the defining torsion angle C1*A*—O11*A*—C12*A*—C13*A* = 176.75 (14) and 176.53 (14)°, respectively. This *anti­periplanar* (180±30°) conformation is similar to those of the parent acids PAA (−175.1°; Kennard *et al.*, 1982 ▸), PFPA [176.0 (6)°; Smith *et al.*, 1992 ▸] and their proton-transfer salts, *e.g.* the ammonium salts of PAA [−177.48 (18)°] and PFPA [−178.98 (17)°] (Smith, 2014 ▸). However, with the 3,5-D and 2,4-D salts, the side-chain conformations are both *synclinal* (90±30°) [−76.5 (2)° in (III) and 72.91 (19)° in (IV)], similar to that in the parent acid 2,4-D (75.2°; Smith *et al.*, 1976 ▸), in the tryptaminium salt of 2,4-D [81.2 (6)°; Smith & Lynch, 2015*a* ▸] and in the 2:1 salt-adduct of 3,5-D with 4,4′-bi­pyridine [−71.6 (3)°; Lynch *et al.*, 2003 ▸]. However, in the tryptaminium salt of 3,5-D (Smith & Lynch, 2015*b* ▸), the ammonium salts of both 2,4-D (Liu *et al.*, 2009 ▸) and 3,5-D (Smith, 2014 ▸), the *anti­periplanar* conformation is found [equivalent torsion angles = −166.5 (3), 172.61 (8) and −171.35 (15)°, respectively].

## Supra­molecular features   

In the crystals of both (I), (III) and (IV), a primary three-centre 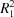
(4) N1*B*—H⋯(O,O′)_carbox­yl_ hydrogen-bonding inter­action is present, with the asymmetry in (I) [N⋯O = 2.7366 (18) and 3.1655 (17) Å] and (IV) [2.683 (2) and 3.115 (2) Å] being significantly greater than that in (III) [2.892 (3) and 2.988 (3) Å] (Tables 1 ▸, 3 ▸ and 4 ▸). With (II), the second N—H⋯O distance is 3.241 (2) Å.

**Figure 5:**
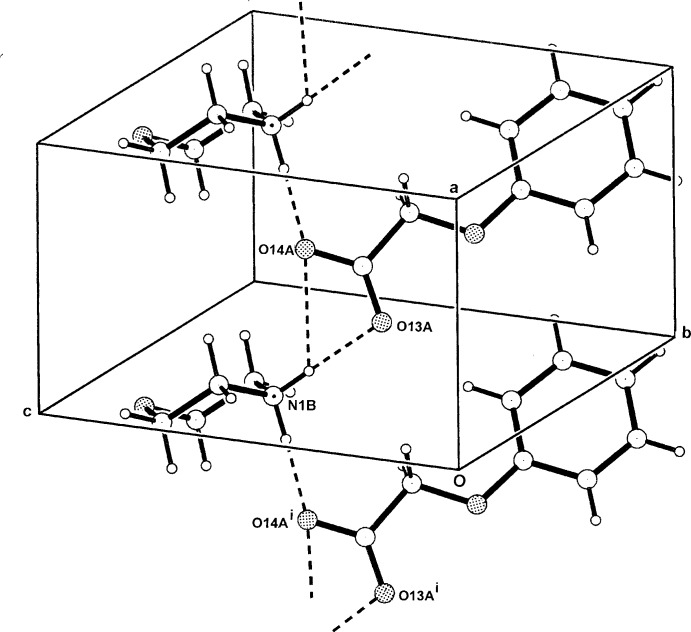
The one-dimensional hydrogen-bonded polymeric structure of (I) extending along *a*. For symmetry codes, see Table 1 ▸.

The hydrogen-bonding extensions involving the second aminium H atom of the cation result in different structures in (I)–(III) compared to that in (IV). With (I)–(III), the primary heterodimers are all extended along *a* through an N1*B*—H⋯O14*A* ^i^ hydrogen bond (Tables 1 ▸–3 ▸ ▸, respectively), into one-dimensional ribbon structures (Figs. 5 ▸–7 ▸ ▸). These ribbon structures provide further examples of the common hydrogen-bonded structure type found among the anhydrous aromatic morpholinium benzoate salts, *e.g.* with salicylic acid (Smith & Lynch, 2015*b* ▸) and with 2-chloro-4-nitro­benzoic acid (Ishida *et al.*, 2001*a* ▸). In both of these examples, helical chains extend along 2_1_screw axes in the crystals. Present also in structures of (I)–(IV) are minor weak inter-unit C—H⋯O inter­actions: in (I), C4*A*—H⋯O4*B* ^ii^ (Table 1 ▸); in (II), C4*A*—H⋯O4*B* ^ii^; C6*B*—H⋯O13*A* ^iii^ (Table 2 ▸): in (III), Cl2*A*—H⋯O13*A* ^ii^ (Table 3 ▸).

**Figure 8:**
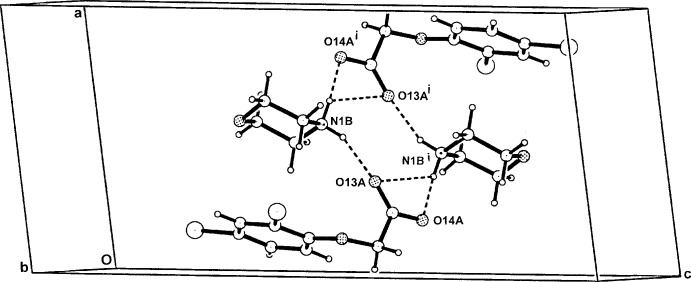
The cyclic hydrogen-bonded hetero­tetra­mer structure of (IV). For symmetry codes, see Table 4 ▸.

In the crystal of (IV), the second N1*B*—H⋯O14*A* ^i^ hydrogen bond generates a centrosymmetric hetero­tetra­meric ring structure [graph set 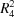
(8)] (Fig. 8 ▸). For symmetry code (i), see Table 4 ▸. This cyclic system typifies the second structure type also found in a number of examples of morpholinium salts with ring-substituted benzoic acids, *e.g.* in the 2-chloro-5-nitro-, 4-chloro-2-nitro-, 4-chloro-3-nitro- and 5-chloro-2-nitro­benzoate series (Ishida *et al.*, 2001*a* ▸,*b* ▸,*c* ▸] and in the 4-amino­salicylate (André *et al.*, 2009 ▸).

Only weak inter-unit C—H⋯O inter­actions to carboxyl or phen­oxy O-atom acceptors are present in (IV) (Table 4 ▸), while no π–π inter­actions are found in any of the structures.

## Synthesis and crystallization   

The title compounds (I)–(IV) were prepared by the dropwise addition of morpholine at room temperature to solutions of phen­oxy­acetic acid (150 mg), (4-fluoro­phen­oxy)acetic (170 mg), (2,4-di­chloro­phen­oxy)acetic acid or (2,4-di­chloro­phen­oxy)acetic acid (220 mg), respectively, in 15 ml of ethanol. Room-temperature evaporation of the solutions gave either colourless plates of (III) or needles of (IV) from which specimens were cleaved for the X-ray analyses. For (I) and (II), the same preparative procedure was employed using phen­oxy­acetic acid or (4-fluoro­phen­oxy)acetic acid but the final oils which resulted after solvent evaporation were redissolved in ethanol, finally giving thin colourless fragile plates of compounds (I) and (II) from which specimens were cleaved for the X-ray analyses.

## Refinement details   

Crystal data, data collection and structure refinement details are given in Table 5 ▸. H atoms were placed in calculated positions (aromatic C—H = 0.95 Å or methyl­ene C—H = 0.99 Å) and were allowed to ride in the refinements, with *U*_iso_(H) = 1.2*U*_eq_(C). The aminium H atoms were located in difference Fourier analyses and were allowed to refine with distance restraints [N—H = 0.90 (2) Å] and *U*_iso_(H) = 1.2*U*_eq_(N).

## Supplementary Material

Crystal structure: contains datablock(s) global, I, II, III, IV. DOI: 10.1107/S2056989015019842/su5227sup1.cif

Structure factors: contains datablock(s) I. DOI: 10.1107/S2056989015019842/su5227Isup2.hkl

Structure factors: contains datablock(s) II. DOI: 10.1107/S2056989015019842/su5227IIsup3.hkl

Structure factors: contains datablock(s) III. DOI: 10.1107/S2056989015019842/su5227IIIsup4.hkl

Structure factors: contains datablock(s) IV. DOI: 10.1107/S2056989015019842/su5227IVsup5.hkl

Supporting information file. DOI: 10.1107/S2056989015019842/su5227Isup6.cml

Supporting information file. DOI: 10.1107/S2056989015019842/su5227IIsup7.cml

Supporting information file. DOI: 10.1107/S2056989015019842/su5227IIIsup8.cml

Supporting information file. DOI: 10.1107/S2056989015019842/su5227IVsup9.cml

CCDC references: 1432389, 1432388, 1432387, 1432386

Additional supporting information:  crystallographic information; 3D view; checkCIF report

## Figures and Tables

**Figure 6:**
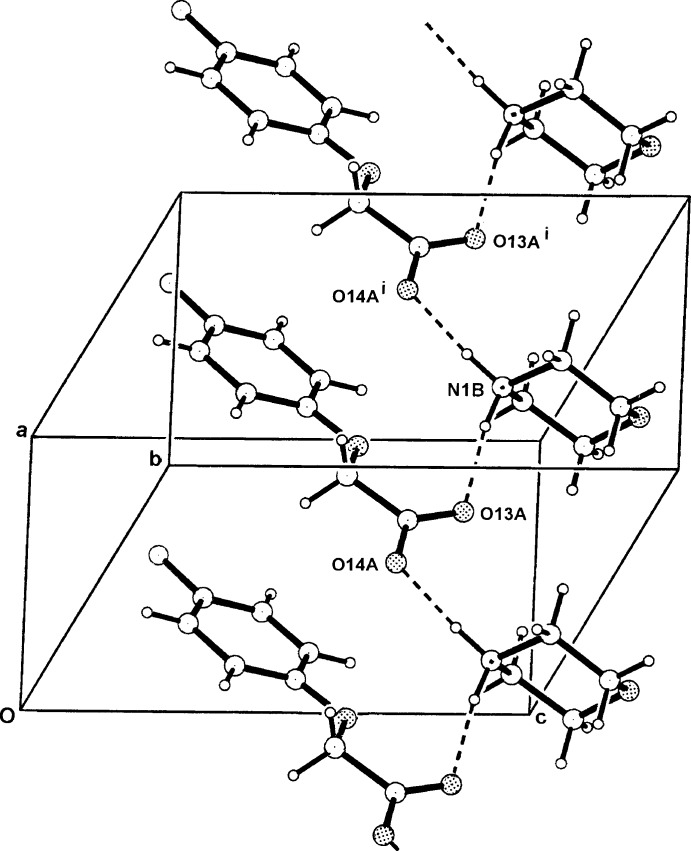
The one-dimensional hydrogen-bonded polymeric structure of (II) extending along *a*. For symmetry codes, see Table 2 ▸.

**Figure 7:**
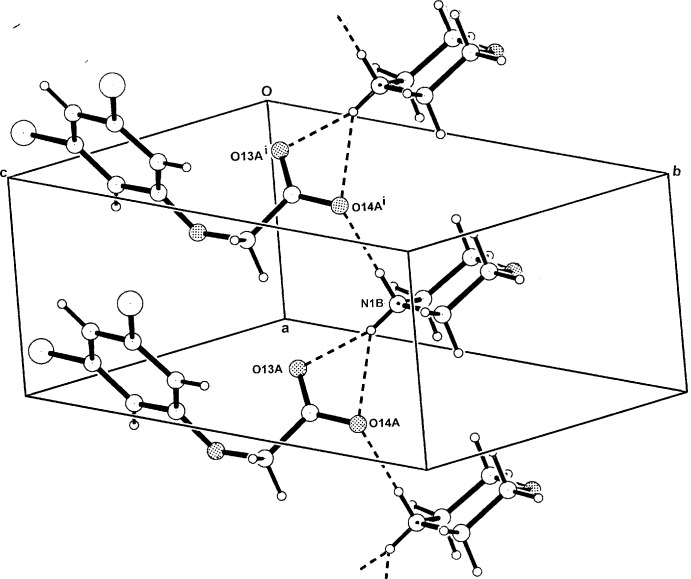
The one-dimensional hydrogen-bonded polymeric structure of (III) extending along *a*. For symmetry codes, see Table 3 ▸

**Table 1:** Hydrogen-bond geometry (Å, °) for (I)

*D*—H⋯*A*	*D*—H	H⋯*A*	*D*⋯*A*	*D*—H⋯*A*
N1*B*—H11*B*⋯O13*A*	0.92 (2)	1.83 (2)	2.7366 (18)	169 (2)
N1*B*—H11*B*⋯O14*A*	0.92 (2)	2.57 (2)	3.1655 (17)	123 (1)
N1*B*—H12*B*⋯O14*A* ^i^	0.95 (1)	1.76 (1)	2.7061 (17)	176 (1)
C4*A*—H4*A*⋯O4*B* ^ii^	0.95	2.59	3.447 (2)	151
C6*B*—H62*B*⋯O13*A* ^iii^	0.99	2.39	3.148 (2)	133

Symmetry codes: (i) 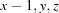; (ii) 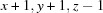; (iii) 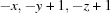.

**Table 2:** Hydrogen-bond geometry (Å, °) for (II)

*D*—H⋯*A*	*D*—H	H⋯*A*	*D*⋯*A*	*D*—H⋯*A*
N1*B*—H11*B*⋯O14*A* ^i^	0.97 (2)	1.76 (2)	2.725 (2)	175 (2)
N1*B*—H12*B*⋯O13*A*	0.94 (2)	1.80 (2)	2.718 (2)	165 (2)
C6*B*—H61*B*⋯O14*A* ^ii^	0.99	2.38	3.188 (2)	138

Symmetry codes: (i) 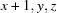; (ii) 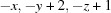.

**Table 3:** Hydrogen-bond geometry (Å, °) for (III)

*D*—H⋯*A*	*D*—H	H⋯*A*	*D*⋯*A*	*D*—H⋯*A*
N1*B*—H11*B*⋯O13*A*	0.88 (2)	2.07 (2)	2.892 (3)	156 (2)
N1*B*—H11*B*⋯O14*A*	0.88 (2)	2.26 (2)	2.988 (3)	141 (2)
N1*B*—H12*B*⋯O14*A* ^i^	0.88 (2)	1.87 (2)	2.737 (3)	170 (2)
C12*A*—H12*A*⋯O13*A* ^ii^	0.99	2.41	3.398 (3)	173

Symmetry codes: (i) 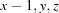; (ii) 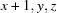.

**Table 4:** Hydrogen-bond geometry (Å, °) for (IV)

*D*—H⋯*A*	*D*—H	H⋯*A*	*D*⋯*A*	*D*—H⋯*A*
N1*B*—H11*B*⋯O13*A* ^i^	0.91 (2)	2.56 (2)	3.115 (2)	120 (1)
N1*B*—H11*B*⋯O14*A* ^i^	0.91 (2)	1.79 (2)	2.683 (2)	169 (2)
N1*B*—H12*B*⋯O13*A*	0.87 (2)	1.92 (2)	2.747 (2)	158 (2)
C12*A*—H12*A*⋯O14*A* ^ii^	0.99	2.50	3.484 (2)	173
C2*B*—H21*B*⋯O11*A* ^iii^	0.99	2.57	3.477 (2)	151
C5*B*—H52*B*⋯O4*B* ^iv^	0.99	2.58	3.489 (3)	153

Symmetry codes: (i) 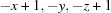; (ii) 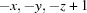; (iii) 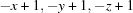; (iv) 
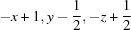
.

**Table 5:** Experimental details

	(I)	(II)	(III)	(IV)
Crystal data				
Chemical formula	C_4_H_10_NO^+^·C_8_H_7_O_3_^−^	C_4_H_10_NO^+^·C_8_H_6_FO_3_^−^	C_4_H_10_NO^+^·C_8_H_5_Cl_2_O_3_^−^	C_4_H_10_NO^+^·C_8_H_5_Cl_2_O_3_^−^
*M*_r_	239.27	257.26	308.15	308.15
Crystal system, space group	Triclinic, *P* 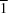	Triclinic*, P* 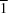	Triclinic, *P* 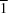	Monoclinic, *P*2_1_/*c*
Temperature (K)	200	200	200	200
*a*, *b*, *c* (Å)	5.7079 (5), 9.7735 (9), 11.3586 (10)	5.7997 (5), 10.2605 (10), 10.4836 (11)	5.1733 (4), 11.3751 (10), 11.7808 (10)	9.3657 (5), 7.1702 (3), 21.1340 (11)
α, β, γ (°)	78.277 (7), 86.171 (7), 77.512 (7)	88.388 (8), 82.792 (8), 80.325 (8)	86.904 (7), 85.106 (7), 77.936 (7)	90, 97.981 (5), 90
*V* (Å^3^)	605.58 (10)	610.11 (10)	675.01 (10)	1405.48 (12)
*Z*	2	2	2	4
Radiation type	Mo *K*α	Mo *K*α	Mo *K*α	Mo *K*α
μ (mm^−1^)	0.10	0.11	0.49	0.47
Crystal size (mm)	0.50 × 0.15 × 0.04	0.50 × 0.25 × 0.05	0.50 × 0.13 × 0.10	0.35 × 0.35 × 0.12

Data collection				
Diffractometer	Oxford Diffraction Gemini-S CCD detector	Oxford Diffraction Gemini-S CCD detector	Oxford Diffraction Gemini-S CCD detector	Oxford Diffraction Gemini-S CCD detector
Absorption correction	Multi-scan (*CrysAlis PRO*; Agilent, 2014 ▸)	Multi-scan (*CrysAlis PRO*; Agilent, 2014 ▸)	Multi-scan (*CrysAlis PRO*; Agilent, 2014 ▸)	Multi-scan (*CrysAlis PRO*; Agilent, 2014 ▸)
*T*_min_, *T*_max_	0.860, 0.980	0.488, 0.980	0.903, 0.989	0.933, 0.980
No. of measured, independent and observed reflections	4172, 2370, 1765 [*I* > 2σ(*I*)]	4984, 2394, 1743 [*I* > 2σ(*I*)]	5616, 2646, 2096 [*I* > 2σ(*I*)]	6400, 2754, 2273 [*I*.2σ(*I*)]
*R*_int_	0.033	0.033	0.027	0.026
(sin θ/λ)_max_ (Å^−1^)	0.617	0.617	0.617	0.617

Refinement				
*R*[*F* ^2^ > 2σ(*F* ^2^)], *wR*(*F* ^2^), *S*	0.048, 0.113, 1.02	0.046, 0.116, 1.04	0.039, 0.091, 1.03	0.038, 0.091, 1.04
No. of reflections	2370	2394	2646	2754
No. of parameters	154	169	178	178
No. of restraints	0	2	2	2
H-atom treatment	H atoms treated by a mixture of independent and constrained refinement	H atoms treated by a mixture of independent and constrained refinement	H atoms treated by a mixture of independent and constrained refinement	H atoms treated by a mixture of independent and constrained refinement
Δρ_max_, Δρ_min_ (e Å^−3^)	0.16, −0.17	0.19, −0.20	0.24, −0.26	0.28, −0.26

Computer programs: *CrysAlis PRO* (Agilent, 2014 ▸), *SIR92* (Altomare *et al.*, 1993 ▸), *SHELXS97* and *SHELXL97* (Sheldrick, 2008 ▸) within *WinGX* (Farrugia, 2012 ▸) and *PLATON* (Spek, 2009 ▸).

## supplementary crystallographic information

### (I) Tetrahydro-2H-1,4-oxazin-4-ium phenoxyacetate Crystal data

**Table:**

C_4_H_10_NO^+^·C_8_H_7_O_3_^−^	*Z* = 2
*M**_r_* = 239.27	*F*(000) = 256
Triclinic, *P*1	*D*_x_ = 1.312 Mg m^−^^3^
Hall symbol: -P 1	Mo *K*α radiation, λ = 0.71073 Å
*a* = 5.7079 (5) Å	Cell parameters from 1029 reflections
*b* = 9.7735 (9) Å	θ = 3.9–28.5°
*c* = 11.3586 (10) Å	µ = 0.10 mm^−^^1^
α = 78.277 (7)°	*T* = 200 K
β = 86.171 (7)°	Plate, colourless
γ = 77.512 (7)°	0.50 × 0.15 × 0.04 mm
*V* = 605.58 (10) Å^3^	

### (I) Tetrahydro-2H-1,4-oxazin-4-ium phenoxyacetate Data collection

**Table:**

Oxford Diffraction Gemini-S CCD-detector diffractometer	2370 independent reflections
Radiation source: Enhance (Mo) X-ray source	1765 reflections with *I* > 2σ(*I*)
Graphite monochromator	*R*_int_ = 0.033
Detector resolution: 16.077 pixels mm^-1^	θ_max_ = 26.0°, θ_min_ = 3.1°
ω scans	*h* = −7→6
Absorption correction: multi-scan (CrysAlis PRO; Agilent, 2014)	*k* = −12→12
*T*_min_ = 0.860, *T*_max_ = 0.980	*l* = −13→13
4172 measured reflections	

### (I) Tetrahydro-2H-1,4-oxazin-4-ium phenoxyacetate Refinement

**Table:**

Refinement on *F*^2^	Primary atom site location: structure-invariant direct methods
Least-squares matrix: full	Secondary atom site location: difference Fourier map
*R*[*F*^2^ > 2σ(*F*^2^)] = 0.048	Hydrogen site location: inferred from neighbouring sites
*wR*(*F*^2^) = 0.113	H atoms treated by a mixture of independent and constrained refinement
*S* = 1.02	*w* = 1/[σ^2^(*F*_o_^2^) + (0.0456*P*)^2^] where *P* = (*F*_o_^2^ + 2*F*_c_^2^)/3
2370 reflections	(Δ/σ)_max_ < 0.001
154 parameters	Δρ_max_ = 0.16 e Å^−^^3^
0 restraints	Δρ_min_ = −0.17 e Å^−^^3^

### (I) Tetrahydro-2H-1,4-oxazin-4-ium phenoxyacetate Special details

**Table:**

Geometry. Bond distances, angles etc. have been calculated using the rounded fractional coordinates. All su's are estimated from the variances of the (full) variance-covariance matrix. The cell esds are taken into account in the estimation of distances, angles and torsion angles
Refinement. Refinement of F^2^ against ALL reflections. The weighted R-factor wR and goodness of fit S are based on F^2^, conventional R-factors R are based on F, with F set to zero for negative F^2^. The threshold expression of F^2^ > 2sigma(F^2^) is used only for calculating R-factors(gt) etc. and is not relevant to the choice of reflections for refinement. R-factors based on F^2^ are statistically about twice as large as those based on F, and R- factors based on ALL data will be even larger.

### (I) Tetrahydro-2H-1,4-oxazin-4-ium phenoxyacetate Fractional atomic coordinates and isotropic or equivalent isotropic displacement parameters (Å^2^)

**Table:**

	*x*	*y*	*z*	*U*_iso_*/*U*_eq_	
O11A	0.3926 (2)	0.81847 (13)	0.38178 (10)	0.0374 (4)	
O13A	0.11992 (19)	0.65034 (13)	0.51716 (10)	0.0358 (4)	
O14A	0.41444 (19)	0.54699 (13)	0.64451 (10)	0.0323 (4)	
C1A	0.5198 (3)	0.91643 (18)	0.32122 (15)	0.0303 (5)	
C2A	0.7157 (3)	0.94974 (19)	0.36542 (16)	0.0346 (6)	
C3A	0.8267 (3)	1.0526 (2)	0.29642 (17)	0.0415 (7)	
C4A	0.7481 (4)	1.1208 (2)	0.18324 (18)	0.0449 (7)	
C5A	0.5549 (4)	1.0854 (2)	0.13875 (17)	0.0432 (7)	
C6A	0.4402 (3)	0.98450 (19)	0.20676 (16)	0.0365 (6)	
C12A	0.4895 (3)	0.73323 (18)	0.49189 (14)	0.0288 (5)	
C13A	0.3264 (3)	0.63578 (18)	0.55411 (14)	0.0268 (5)	
O4B	−0.1545 (2)	0.39037 (15)	0.94974 (10)	0.0445 (5)	
N1B	−0.1183 (2)	0.50356 (15)	0.70035 (12)	0.0270 (4)	
C2B	−0.1277 (3)	0.59359 (19)	0.79155 (14)	0.0322 (6)	
C3B	−0.2632 (3)	0.5347 (2)	0.90159 (15)	0.0386 (6)	
C5B	−0.1515 (4)	0.3030 (2)	0.86300 (16)	0.0410 (7)	
C6B	−0.0136 (3)	0.35172 (19)	0.75076 (15)	0.0349 (6)	
H2A	0.77390	0.90240	0.44270	0.0420*	
H3A	0.95950	1.07660	0.32770	0.0500*	
H4A	0.82570	1.19120	0.13660	0.0540*	
H5A	0.50030	1.13090	0.06040	0.0520*	
H6A	0.30650	0.96140	0.17550	0.0440*	
H11A	0.51320	0.79650	0.54600	0.0350*	
H12A	0.64820	0.67470	0.47530	0.0350*	
H11B	−0.032 (3)	0.5424 (18)	0.6353 (13)	0.0320*	
H12B	−0.280 (2)	0.5164 (19)	0.6782 (13)	0.0320*	
H21B	−0.20870	0.69300	0.75840	0.0390*	
H22B	0.03720	0.59430	0.81310	0.0390*	
H31B	−0.26710	0.59360	0.96330	0.0460*	
H32B	−0.43070	0.53980	0.88040	0.0460*	
H51B	−0.31840	0.30590	0.84220	0.0490*	
H52B	−0.07770	0.20290	0.89820	0.0490*	
H61B	0.15700	0.34140	0.76960	0.0420*	
H62B	−0.02120	0.29200	0.69100	0.0420*	

### (I) Tetrahydro-2H-1,4-oxazin-4-ium phenoxyacetate Atomic displacement parameters (Å^2^)

**Table:**

	*U*^11^	*U*^22^	*U*^33^	*U*^12^	*U*^13^	*U*^23^
O11A	0.0348 (7)	0.0363 (8)	0.0397 (7)	−0.0186 (6)	−0.0046 (5)	0.0088 (6)
O13A	0.0252 (7)	0.0472 (9)	0.0342 (7)	−0.0162 (6)	−0.0014 (5)	0.0031 (6)
O14A	0.0270 (6)	0.0366 (8)	0.0316 (7)	−0.0121 (6)	−0.0004 (5)	0.0030 (6)
C1A	0.0299 (9)	0.0232 (9)	0.0364 (10)	−0.0081 (8)	0.0070 (8)	−0.0021 (8)
C2A	0.0351 (10)	0.0320 (11)	0.0373 (10)	−0.0128 (8)	0.0028 (8)	−0.0028 (8)
C3A	0.0387 (11)	0.0391 (12)	0.0509 (12)	−0.0201 (9)	0.0074 (9)	−0.0086 (10)
C4A	0.0515 (12)	0.0360 (12)	0.0487 (12)	−0.0222 (10)	0.0140 (10)	−0.0025 (10)
C5A	0.0545 (12)	0.0326 (11)	0.0387 (11)	−0.0117 (10)	0.0037 (9)	0.0030 (9)
C6A	0.0378 (10)	0.0307 (11)	0.0405 (10)	−0.0105 (9)	−0.0012 (8)	−0.0018 (8)
C12A	0.0255 (9)	0.0281 (10)	0.0328 (9)	−0.0101 (8)	0.0001 (7)	−0.0012 (8)
C13A	0.0257 (9)	0.0290 (10)	0.0273 (9)	−0.0093 (8)	0.0044 (7)	−0.0071 (8)
O4B	0.0641 (9)	0.0436 (9)	0.0267 (7)	−0.0196 (7)	−0.0014 (6)	0.0001 (6)
N1B	0.0239 (7)	0.0331 (9)	0.0247 (7)	−0.0120 (7)	0.0021 (6)	−0.0015 (6)
C2B	0.0333 (10)	0.0317 (10)	0.0342 (10)	−0.0119 (8)	0.0013 (8)	−0.0073 (8)
C3B	0.0458 (11)	0.0422 (12)	0.0295 (10)	−0.0137 (9)	0.0061 (8)	−0.0083 (9)
C5B	0.0577 (13)	0.0309 (11)	0.0363 (10)	−0.0180 (9)	−0.0055 (9)	−0.0003 (8)
C6B	0.0366 (10)	0.0313 (11)	0.0356 (10)	−0.0045 (8)	−0.0037 (8)	−0.0057 (8)

### (I) Tetrahydro-2H-1,4-oxazin-4-ium phenoxyacetate Geometric parameters (Å, º)

**Table:**

O11A—C1A	1.372 (2)	C2A—H2A	0.9500
O11A—C12A	1.426 (2)	C3A—H3A	0.9500
O13A—C13A	1.247 (2)	C4A—H4A	0.9500
O14A—C13A	1.256 (2)	C5A—H5A	0.9500
O4B—C5B	1.426 (2)	C6A—H6A	0.9500
O4B—C3B	1.424 (2)	C12A—H11A	0.9900
N1B—C6B	1.485 (2)	C12A—H12A	0.9900
N1B—C2B	1.481 (2)	C2B—C3B	1.504 (2)
N1B—H12B	0.948 (12)	C5B—C6B	1.501 (3)
N1B—H11B	0.923 (16)	C2B—H21B	0.9900
C1A—C6A	1.391 (2)	C2B—H22B	0.9900
C1A—C2A	1.381 (2)	C3B—H31B	0.9900
C2A—C3A	1.384 (3)	C3B—H32B	0.9900
C3A—C4A	1.378 (3)	C5B—H51B	0.9900
C4A—C5A	1.379 (3)	C5B—H52B	0.9900
C5A—C6A	1.378 (3)	C6B—H61B	0.9900
C12A—C13A	1.515 (2)	C6B—H62B	0.9900
			
C1A—O11A—C12A	116.65 (13)	C13A—C12A—H11A	109.00
C3B—O4B—C5B	110.28 (13)	O11A—C12A—H11A	109.00
C2B—N1B—C6B	110.91 (13)	C13A—C12A—H12A	109.00
C6B—N1B—H11B	113.4 (11)	O11A—C12A—H12A	109.00
C2B—N1B—H12B	104.8 (10)	H11A—C12A—H12A	108.00
H11B—N1B—H12B	109.0 (14)	N1B—C2B—C3B	109.23 (14)
C2B—N1B—H11B	106.3 (10)	O4B—C3B—C2B	111.23 (14)
C6B—N1B—H12B	111.9 (11)	O4B—C5B—C6B	111.72 (16)
O11A—C1A—C6A	115.56 (15)	N1B—C6B—C5B	109.35 (14)
C2A—C1A—C6A	119.48 (16)	N1B—C2B—H21B	110.00
O11A—C1A—C2A	124.96 (15)	N1B—C2B—H22B	110.00
C1A—C2A—C3A	119.58 (16)	C3B—C2B—H21B	110.00
C2A—C3A—C4A	121.20 (18)	C3B—C2B—H22B	110.00
C3A—C4A—C5A	118.97 (19)	H21B—C2B—H22B	108.00
C4A—C5A—C6A	120.65 (18)	O4B—C3B—H31B	109.00
C1A—C6A—C5A	120.10 (17)	O4B—C3B—H32B	109.00
O11A—C12A—C13A	111.82 (14)	C2B—C3B—H31B	109.00
O13A—C13A—O14A	125.13 (16)	C2B—C3B—H32B	109.00
O13A—C13A—C12A	120.24 (14)	H31B—C3B—H32B	108.00
O14A—C13A—C12A	114.59 (15)	O4B—C5B—H51B	109.00
C3A—C2A—H2A	120.00	O4B—C5B—H52B	109.00
C1A—C2A—H2A	120.00	C6B—C5B—H51B	109.00
C4A—C3A—H3A	119.00	C6B—C5B—H52B	109.00
C2A—C3A—H3A	119.00	H51B—C5B—H52B	108.00
C5A—C4A—H4A	121.00	N1B—C6B—H61B	110.00
C3A—C4A—H4A	121.00	N1B—C6B—H62B	110.00
C4A—C5A—H5A	120.00	C5B—C6B—H61B	110.00
C6A—C5A—H5A	120.00	C5B—C6B—H62B	110.00
C5A—C6A—H6A	120.00	H61B—C6B—H62B	108.00
C1A—C6A—H6A	120.00		
			
C12A—O11A—C1A—C2A	−8.1 (2)	O11A—C1A—C2A—C3A	−178.84 (17)
C12A—O11A—C1A—C6A	171.64 (15)	C1A—C2A—C3A—C4A	−1.2 (3)
C1A—O11A—C12A—C13A	176.53 (14)	C2A—C3A—C4A—C5A	0.1 (3)
C5B—O4B—C3B—C2B	−60.23 (18)	C3A—C4A—C5A—C6A	0.7 (3)
C3B—O4B—C5B—C6B	59.8 (2)	C4A—C5A—C6A—C1A	−0.4 (3)
C6B—N1B—C2B—C3B	−55.34 (17)	O11A—C12A—C13A—O14A	172.45 (14)
C2B—N1B—C6B—C5B	54.74 (18)	O11A—C12A—C13A—O13A	−9.8 (2)
C6A—C1A—C2A—C3A	1.5 (3)	N1B—C2B—C3B—O4B	58.00 (18)
O11A—C1A—C6A—C5A	179.61 (16)	O4B—C5B—C6B—N1B	−56.8 (2)
C2A—C1A—C6A—C5A	−0.7 (3)		

### (I) Tetrahydro-2H-1,4-oxazin-4-ium phenoxyacetate Hydrogen-bond geometry (Å, º)

**Table:**

*D*—H···*A*	*D*—H	H···*A*	*D*···*A*	*D*—H···*A*
N1*B*—H11*B*···O13*A*	0.92 (2)	1.83 (2)	2.7366 (18)	169 (2)
N1*B*—H11*B*···O14*A*	0.92 (2)	2.57 (2)	3.1655 (17)	123 (1)
N1*B*—H12*B*···O14*A*^i^	0.95 (1)	1.76 (1)	2.7061 (17)	176 (1)
C4*A*—H4*A*···O4*B*^ii^	0.95	2.59	3.447 (2)	151
C6*B*—H62*B*···O13*A*^iii^	0.99	2.39	3.148 (2)	133

Symmetry codes: (i) *x*−1, *y*, *z*; (ii) *x*+1, *y*+1, *z*−1; (iii) −*x*, −*y*+1, −*z*+1.

### (II) Tetrahydro-2H-1,4-oxazin-4-ium (4-fluorophenoxy)acetate Crystal data

**Table:**

C_4_H_10_NO^+^·C_8_H_6_FO_3_^−^	*Z* = 2
*M**_r_* = 257.26	*F*(000) = 272
Triclinic, *P*1	*D*_x_ = 1.400 Mg m^−^^3^
Hall symbol: -P 1	Mo *K*α radiation, λ = 0.71073 Å
*a* = 5.7997 (5) Å	Cell parameters from 1163 reflections
*b* = 10.2605 (10) Å	θ = 4.0–28.4°
*c* = 10.4836 (11) Å	µ = 0.11 mm^−^^1^
α = 88.388 (8)°	*T* = 200 K
β = 82.792 (8)°	Plate, colourless
γ = 80.325 (8)°	0.50 × 0.25 × 0.05 mm
*V* = 610.11 (10) Å^3^	

### (II) Tetrahydro-2H-1,4-oxazin-4-ium (4-fluorophenoxy)acetate Data collection

**Table:**

Oxford Diffraction Gemini-S CCD-detector diffractometer	2394 independent reflections
Radiation source: fine-focus sealed tube	1743 reflections with *I* > 2σ(*I*)
Graphite monochromator	*R*_int_ = 0.033
Detector resolution: 16.077 pixels mm^-1^	θ_max_ = 26.0°, θ_min_ = 3.6°
ω scans	*h* = −7→6
Absorption correction: multi-scan (CrysAlis PRO; Agilent, 2014)	*k* = −10→12
*T*_min_ = 0.488, *T*_max_ = 0.980	*l* = −12→12
4984 measured reflections	

### (II) Tetrahydro-2H-1,4-oxazin-4-ium (4-fluorophenoxy)acetate Refinement

**Table:**

Refinement on *F*^2^	Primary atom site location: structure-invariant direct methods
Least-squares matrix: full	Secondary atom site location: difference Fourier map
*R*[*F*^2^ > 2σ(*F*^2^)] = 0.046	Hydrogen site location: inferred from neighbouring sites
*wR*(*F*^2^) = 0.116	H atoms treated by a mixture of independent and constrained refinement
*S* = 1.04	*w* = 1/[σ^2^(*F*_o_^2^) + (0.046*P*)^2^ + 0.0267*P*] where *P* = (*F*_o_^2^ + 2*F*_c_^2^)/3
2394 reflections	(Δ/σ)_max_ < 0.001
169 parameters	Δρ_max_ = 0.19 e Å^−^^3^
2 restraints	Δρ_min_ = −0.20 e Å^−^^3^

### (II) Tetrahydro-2H-1,4-oxazin-4-ium (4-fluorophenoxy)acetate Special details

**Table:**

Geometry. Bond distances, angles etc. have been calculated using the rounded fractional coordinates. All su's are estimated from the variances of the (full) variance-covariance matrix. The cell esds are taken into account in the estimation of distances, angles and torsion angles
Refinement. Refinement of F^2^ against ALL reflections. The weighted R-factor wR and goodness of fit S are based on F^2^, conventional R-factors R are based on F, with F set to zero for negative F^2^. The threshold expression of F^2^ > 2sigma(F^2^) is used only for calculating R-factors(gt) etc. and is not relevant to the choice of reflections for refinement. R-factors based on F^2^ are statistically about twice as large as those based on F, and R- factors based on ALL data will be even larger.

### (II) Tetrahydro-2H-1,4-oxazin-4-ium (4-fluorophenoxy)acetate Fractional atomic coordinates and isotropic or equivalent isotropic displacement parameters (Å^2^)

**Table:**

	*x*	*y*	*z*	*U*_iso_*/*U*_eq_	
F4A	1.10905 (19)	0.50899 (12)	0.11938 (12)	0.0518 (4)	
O11A	0.3317 (2)	0.71651 (12)	0.44716 (12)	0.0328 (4)	
O13A	−0.0039 (2)	0.82589 (13)	0.62958 (12)	0.0331 (4)	
O14A	−0.2493 (2)	0.88910 (13)	0.48447 (12)	0.0350 (4)	
C1A	0.5169 (3)	0.66778 (16)	0.35718 (18)	0.0255 (6)	
C2A	0.5357 (3)	0.69979 (18)	0.22788 (18)	0.0300 (6)	
C3A	0.7372 (3)	0.64655 (19)	0.14698 (19)	0.0357 (6)	
C4A	0.9096 (3)	0.56021 (18)	0.1981 (2)	0.0331 (6)	
C5A	0.8925 (3)	0.52405 (18)	0.3247 (2)	0.0333 (6)	
C6A	0.6948 (3)	0.57908 (18)	0.40512 (19)	0.0308 (6)	
C12A	0.1408 (3)	0.80714 (17)	0.40429 (17)	0.0263 (6)	
C13A	−0.0514 (3)	0.84287 (16)	0.51649 (18)	0.0249 (6)	
O4B	0.3300 (2)	0.84416 (14)	0.96211 (13)	0.0458 (5)	
N1B	0.4282 (3)	0.85297 (15)	0.68920 (15)	0.0288 (5)	
C2B	0.4898 (3)	0.72899 (19)	0.76326 (19)	0.0357 (7)	
C3B	0.3244 (4)	0.7319 (2)	0.8863 (2)	0.0415 (7)	
C5B	0.2614 (4)	0.9620 (2)	0.8921 (2)	0.0425 (7)	
C6B	0.4214 (3)	0.96981 (18)	0.76981 (19)	0.0329 (6)	
H2A	0.41190	0.75780	0.19420	0.0360*	
H3A	0.75460	0.66960	0.05830	0.0430*	
H5A	1.01350	0.46260	0.35680	0.0400*	
H6A	0.68030	0.55610	0.49380	0.0370*	
H11A	0.07620	0.76630	0.33510	0.0320*	
H12A	0.19810	0.88810	0.36920	0.0320*	
H11B	0.541 (3)	0.861 (2)	0.6143 (17)	0.0500*	
H12B	0.283 (3)	0.855 (2)	0.6580 (19)	0.0500*	
H21B	0.47750	0.65180	0.71140	0.0430*	
H22B	0.65420	0.72040	0.78310	0.0430*	
H31B	0.36880	0.65030	0.93650	0.0500*	
H32B	0.16190	0.73360	0.86570	0.0500*	
H51B	0.26350	1.03940	0.94600	0.0510*	
H52B	0.09820	0.96510	0.87220	0.0510*	
H61B	0.36500	1.05120	0.72200	0.0390*	
H62B	0.58220	0.97440	0.78970	0.0390*	

### (II) Tetrahydro-2H-1,4-oxazin-4-ium (4-fluorophenoxy)acetate Atomic displacement parameters (Å^2^)

**Table:**

	*U*^11^	*U*^22^	*U*^33^	*U*^12^	*U*^13^	*U*^23^
F4A	0.0376 (7)	0.0649 (8)	0.0429 (8)	0.0137 (6)	0.0067 (6)	−0.0140 (6)
O11A	0.0251 (6)	0.0409 (8)	0.0265 (8)	0.0079 (5)	0.0010 (5)	0.0031 (6)
O13A	0.0230 (6)	0.0513 (8)	0.0237 (8)	−0.0042 (6)	−0.0012 (6)	0.0015 (6)
O14A	0.0216 (7)	0.0497 (8)	0.0300 (8)	0.0041 (6)	−0.0036 (6)	0.0026 (6)
C1A	0.0231 (9)	0.0256 (9)	0.0259 (11)	−0.0003 (7)	−0.0003 (8)	−0.0031 (8)
C2A	0.0297 (10)	0.0304 (10)	0.0270 (11)	0.0040 (8)	−0.0045 (8)	−0.0024 (8)
C3A	0.0379 (11)	0.0405 (11)	0.0244 (11)	0.0025 (9)	0.0015 (9)	−0.0043 (9)
C4A	0.0265 (10)	0.0342 (10)	0.0346 (13)	0.0025 (8)	0.0039 (8)	−0.0122 (9)
C5A	0.0253 (9)	0.0337 (10)	0.0383 (13)	0.0040 (8)	−0.0053 (9)	−0.0006 (9)
C6A	0.0287 (10)	0.0344 (10)	0.0275 (11)	−0.0018 (8)	−0.0018 (8)	0.0043 (9)
C12A	0.0237 (9)	0.0273 (9)	0.0259 (11)	0.0011 (7)	−0.0022 (8)	0.0005 (8)
C13A	0.0224 (9)	0.0261 (9)	0.0266 (11)	−0.0053 (7)	−0.0023 (8)	−0.0003 (8)
O4B	0.0628 (10)	0.0521 (9)	0.0227 (8)	−0.0119 (7)	−0.0039 (7)	0.0049 (7)
N1B	0.0222 (8)	0.0415 (9)	0.0217 (9)	−0.0032 (7)	−0.0014 (7)	0.0008 (7)
C2B	0.0345 (10)	0.0336 (11)	0.0384 (13)	−0.0030 (8)	−0.0062 (9)	0.0006 (9)
C3B	0.0494 (13)	0.0433 (12)	0.0339 (13)	−0.0160 (10)	−0.0043 (10)	0.0084 (10)
C5B	0.0488 (12)	0.0450 (13)	0.0300 (13)	−0.0015 (10)	0.0015 (10)	−0.0025 (10)
C6B	0.0337 (10)	0.0346 (11)	0.0298 (12)	−0.0034 (8)	−0.0057 (9)	0.0034 (9)

### (II) Tetrahydro-2H-1,4-oxazin-4-ium (4-fluorophenoxy)acetate Geometric parameters (Å, º)

**Table:**

F4A—C4A	1.370 (2)	C12A—C13A	1.523 (3)
O11A—C1A	1.373 (2)	C2A—H2A	0.9500
O11A—C12A	1.431 (2)	C3A—H3A	0.9500
O13A—C13A	1.251 (2)	C5A—H5A	0.9500
O14A—C13A	1.249 (2)	C6A—H6A	0.9500
O4B—C5B	1.422 (2)	C12A—H11A	0.9900
O4B—C3B	1.425 (2)	C12A—H12A	0.9900
N1B—C6B	1.478 (2)	C2B—C3B	1.506 (3)
N1B—C2B	1.487 (2)	C5B—C6B	1.494 (3)
N1B—H12B	0.938 (18)	C2B—H21B	0.9900
N1B—H11B	0.968 (18)	C2B—H22B	0.9900
C1A—C6A	1.391 (3)	C3B—H31B	0.9900
C1A—C2A	1.381 (3)	C3B—H32B	0.9900
C2A—C3A	1.395 (3)	C5B—H51B	0.9900
C3A—C4A	1.372 (3)	C5B—H52B	0.9900
C4A—C5A	1.364 (3)	C6B—H61B	0.9900
C5A—C6A	1.382 (3)	C6B—H62B	0.9900
			
C1A—O11A—C12A	117.83 (14)	C13A—C12A—H11A	110.00
C3B—O4B—C5B	109.82 (15)	O11A—C12A—H11A	110.00
C2B—N1B—C6B	110.58 (15)	C13A—C12A—H12A	110.00
C6B—N1B—H11B	106.7 (12)	O11A—C12A—H12A	110.00
C2B—N1B—H12B	110.3 (12)	H11A—C12A—H12A	108.00
H11B—N1B—H12B	105.8 (16)	N1B—C2B—C3B	109.48 (16)
C2B—N1B—H11B	112.8 (12)	O4B—C3B—C2B	111.75 (17)
C6B—N1B—H12B	110.5 (12)	O4B—C5B—C6B	111.70 (17)
O11A—C1A—C6A	114.80 (16)	N1B—C6B—C5B	110.52 (15)
C2A—C1A—C6A	119.88 (17)	N1B—C2B—H21B	110.00
O11A—C1A—C2A	125.33 (16)	N1B—C2B—H22B	110.00
C1A—C2A—C3A	119.71 (17)	C3B—C2B—H21B	110.00
C2A—C3A—C4A	118.64 (18)	C3B—C2B—H22B	110.00
C3A—C4A—C5A	122.83 (18)	H21B—C2B—H22B	108.00
F4A—C4A—C5A	118.33 (16)	O4B—C3B—H31B	109.00
F4A—C4A—C3A	118.84 (18)	O4B—C3B—H32B	109.00
C4A—C5A—C6A	118.38 (17)	C2B—C3B—H31B	109.00
C1A—C6A—C5A	120.53 (18)	C2B—C3B—H32B	109.00
O11A—C12A—C13A	109.56 (14)	H31B—C3B—H32B	108.00
O13A—C13A—O14A	125.43 (17)	O4B—C5B—H51B	109.00
O14A—C13A—C12A	114.50 (16)	O4B—C5B—H52B	109.00
O13A—C13A—C12A	120.06 (15)	C6B—C5B—H51B	109.00
C3A—C2A—H2A	120.00	C6B—C5B—H52B	109.00
C1A—C2A—H2A	120.00	H51B—C5B—H52B	108.00
C4A—C3A—H3A	121.00	N1B—C6B—H61B	110.00
C2A—C3A—H3A	121.00	N1B—C6B—H62B	110.00
C4A—C5A—H5A	121.00	C5B—C6B—H61B	110.00
C6A—C5A—H5A	121.00	C5B—C6B—H62B	110.00
C5A—C6A—H6A	120.00	H61B—C6B—H62B	108.00
C1A—C6A—H6A	120.00		
			
C12A—O11A—C1A—C2A	0.5 (2)	C1A—C2A—C3A—C4A	1.8 (3)
C12A—O11A—C1A—C6A	−179.26 (15)	C2A—C3A—C4A—F4A	−179.19 (16)
C1A—O11A—C12A—C13A	176.75 (14)	C2A—C3A—C4A—C5A	0.0 (3)
C3B—O4B—C5B—C6B	−59.8 (2)	C3A—C4A—C5A—C6A	−1.4 (3)
C5B—O4B—C3B—C2B	60.3 (2)	F4A—C4A—C5A—C6A	177.88 (16)
C6B—N1B—C2B—C3B	53.5 (2)	C4A—C5A—C6A—C1A	0.8 (3)
C2B—N1B—C6B—C5B	−53.7 (2)	O11A—C12A—C13A—O14A	−160.39 (14)
C6A—C1A—C2A—C3A	−2.3 (3)	O11A—C12A—C13A—O13A	20.1 (2)
O11A—C1A—C2A—C3A	177.92 (16)	N1B—C2B—C3B—O4B	−57.4 (2)
C2A—C1A—C6A—C5A	1.0 (3)	O4B—C5B—C6B—N1B	57.1 (2)
O11A—C1A—C6A—C5A	−179.22 (16)		

### (II) Tetrahydro-2H-1,4-oxazin-4-ium (4-fluorophenoxy)acetate Hydrogen-bond geometry (Å, º)

**Table:**

*D*—H···*A*	*D*—H	H···*A*	*D*···*A*	*D*—H···*A*
N1*B*—H11*B*···O14*A*^i^	0.97 (2)	1.76 (2)	2.725 (2)	175 (2)
N1*B*—H12*B*···O13*A*	0.94 (2)	1.80 (2)	2.718 (2)	165 (2)
C6*B*—H61*B*···O14*A*^ii^	0.99	2.38	3.188 (2)	138

Symmetry codes: (i) *x*+1, *y*, *z*; (ii) −*x*, −*y*+2, −*z*+1.

### (III) Tetrahydro-2H-1,4-oxazin-4-ium (3,5-dichlorophenoxy)acetate Crystal data

**Table:**

C_4_H_10_NO^+^·C_8_H_5_Cl_2_O_3_^−^	*Z* = 2
*M**_r_* = 308.15	*F*(000) = 320
Triclinic, *P*1	*D*_x_ = 1.516 Mg m^−^^3^
Hall symbol: -P 1	Mo *K*α radiation, λ = 0.71073 Å
*a* = 5.1733 (4) Å	Cell parameters from 1520 reflections
*b* = 11.3751 (10) Å	θ = 3.7–27.8°
*c* = 11.7808 (10) Å	µ = 0.49 mm^−^^1^
α = 86.904 (7)°	*T* = 200 K
β = 85.106 (7)°	Needle, colourless
γ = 77.936 (7)°	0.50 × 0.13 × 0.10 mm
*V* = 675.01 (10) Å^3^	

### (III) Tetrahydro-2H-1,4-oxazin-4-ium (3,5-dichlorophenoxy)acetate Data collection

**Table:**

Oxford Diffraction Gemini-S CCD-detector diffractometer	2646 independent reflections
Radiation source: fine-focus sealed tube	2096 reflections with *I* > 2σ(*I*)
Graphite monochromator	*R*_int_ = 0.027
Detector resolution: 16.077 pixels mm^-1^	θ_max_ = 26.0°, θ_min_ = 3.5°
ω scans	*h* = −6→6
Absorption correction: multi-scan (CrysAlis PRO; Agilent, 2014)	*k* = −14→12
*T*_min_ = 0.903, *T*_max_ = 0.989	*l* = −14→14
5616 measured reflections	

### (III) Tetrahydro-2H-1,4-oxazin-4-ium (3,5-dichlorophenoxy)acetate Refinement

**Table:**

Refinement on *F*^2^	Primary atom site location: structure-invariant direct methods
Least-squares matrix: full	Secondary atom site location: difference Fourier map
*R*[*F*^2^ > 2σ(*F*^2^)] = 0.039	Hydrogen site location: inferred from neighbouring sites
*wR*(*F*^2^) = 0.091	H atoms treated by a mixture of independent and constrained refinement
*S* = 1.03	*w* = 1/[σ^2^(*F*_o_^2^) + (0.0328*P*)^2^ + 0.273*P*] where *P* = (*F*_o_^2^ + 2*F*_c_^2^)/3
2646 reflections	(Δ/σ)_max_ = 0.001
178 parameters	Δρ_max_ = 0.24 e Å^−^^3^
2 restraints	Δρ_min_ = −0.26 e Å^−^^3^

### (III) Tetrahydro-2H-1,4-oxazin-4-ium (3,5-dichlorophenoxy)acetate Special details

**Table:**

Geometry. Bond distances, angles etc. have been calculated using the rounded fractional coordinates. All su's are estimated from the variances of the (full) variance-covariance matrix. The cell esds are taken into account in the estimation of distances, angles and torsion angles
Refinement. Refinement of F^2^ against ALL reflections. The weighted R-factor wR and goodness of fit S are based on F^2^, conventional R-factors R are based on F, with F set to zero for negative F^2^. The threshold expression of F^2^ > 2sigma(F^2^) is used only for calculating R-factors(gt) etc. and is not relevant to the choice of reflections for refinement. R-factors based on F^2^ are statistically about twice as large as those based on F, and R- factors based on ALL data will be even larger.

### (III) Tetrahydro-2H-1,4-oxazin-4-ium (3,5-dichlorophenoxy)acetate Fractional atomic coordinates and isotropic or equivalent isotropic displacement parameters (Å^2^)

**Table:**

	*x*	*y*	*z*	*U*_iso_*/*U*_eq_	
Cl3A	0.43721 (11)	0.33427 (5)	1.07395 (5)	0.0409 (2)	
Cl5A	0.87160 (12)	−0.05932 (5)	0.83964 (5)	0.0423 (2)	
O11A	1.2226 (3)	0.31888 (13)	0.77458 (13)	0.0356 (5)	
O13A	0.8276 (3)	0.47790 (19)	0.67505 (15)	0.0575 (7)	
O14A	1.0229 (3)	0.63014 (15)	0.69348 (14)	0.0476 (6)	
C1A	1.0356 (4)	0.26801 (19)	0.83573 (17)	0.0271 (6)	
C2A	0.8470 (4)	0.32703 (19)	0.91505 (17)	0.0281 (6)	
C3A	0.6719 (4)	0.26282 (19)	0.97100 (17)	0.0279 (6)	
C4A	0.6730 (4)	0.14542 (19)	0.94986 (17)	0.0307 (7)	
C5A	0.8641 (4)	0.08971 (19)	0.87030 (18)	0.0303 (7)	
C6A	1.0479 (4)	0.14815 (19)	0.81392 (18)	0.0300 (7)	
C12A	1.2129 (4)	0.44437 (19)	0.77898 (19)	0.0306 (7)	
C13A	1.0030 (4)	0.5221 (2)	0.70886 (18)	0.0335 (7)	
O4B	0.3249 (3)	0.88990 (15)	0.45028 (14)	0.0502 (6)	
N1B	0.4795 (4)	0.70096 (19)	0.61100 (17)	0.0416 (7)	
C2B	0.5070 (5)	0.6814 (2)	0.48655 (19)	0.0395 (8)	
C3B	0.3059 (4)	0.7719 (2)	0.4277 (2)	0.0418 (8)	
C5B	0.4761 (4)	0.8273 (2)	0.6354 (2)	0.0415 (8)	
C6B	0.2754 (5)	0.9090 (2)	0.5686 (2)	0.0466 (8)	
H2A	0.83820	0.40890	0.93060	0.0340*	
H4A	0.54740	0.10420	0.98840	0.0370*	
H6A	1.18090	0.10720	0.76100	0.0360*	
H12A	1.38860	0.46070	0.75130	0.0370*	
H13A	1.17790	0.46790	0.85940	0.0370*	
H11B	0.607 (4)	0.648 (2)	0.641 (2)	0.0560*	
H12B	0.332 (4)	0.684 (2)	0.644 (2)	0.0560*	
H21B	0.48270	0.59940	0.47290	0.0470*	
H22B	0.68720	0.68820	0.45520	0.0470*	
H31B	0.33260	0.76060	0.34450	0.0500*	
H32B	0.12620	0.75950	0.45370	0.0500*	
H51B	0.65360	0.84550	0.61490	0.0500*	
H52B	0.43210	0.84010	0.71790	0.0500*	
H61B	0.09660	0.89510	0.59410	0.0560*	
H62B	0.27880	0.99360	0.58300	0.0560*	

### (III) Tetrahydro-2H-1,4-oxazin-4-ium (3,5-dichlorophenoxy)acetate Atomic displacement parameters (Å^2^)

**Table:**

	*U*^11^	*U*^22^	*U*^33^	*U*^12^	*U*^13^	*U*^23^
Cl3A	0.0453 (3)	0.0386 (3)	0.0341 (3)	−0.0032 (3)	0.0093 (2)	−0.0012 (2)
Cl5A	0.0563 (4)	0.0308 (3)	0.0431 (3)	−0.0179 (3)	0.0037 (3)	−0.0068 (2)
O11A	0.0296 (8)	0.0266 (8)	0.0495 (10)	−0.0083 (6)	0.0073 (7)	0.0017 (7)
O13A	0.0363 (10)	0.0903 (15)	0.0527 (11)	−0.0302 (10)	−0.0208 (8)	0.0323 (10)
O14A	0.0523 (10)	0.0345 (10)	0.0491 (10)	0.0042 (8)	−0.0036 (8)	0.0083 (8)
C1A	0.0251 (10)	0.0283 (11)	0.0291 (11)	−0.0076 (9)	−0.0058 (9)	0.0029 (9)
C2A	0.0310 (11)	0.0228 (11)	0.0310 (11)	−0.0054 (9)	−0.0070 (9)	0.0017 (9)
C3A	0.0291 (11)	0.0308 (12)	0.0226 (10)	−0.0040 (9)	−0.0026 (8)	0.0026 (9)
C4A	0.0329 (12)	0.0330 (12)	0.0274 (11)	−0.0113 (9)	−0.0018 (9)	0.0046 (9)
C5A	0.0375 (12)	0.0256 (11)	0.0296 (11)	−0.0085 (9)	−0.0083 (9)	0.0008 (9)
C6A	0.0296 (11)	0.0303 (12)	0.0292 (11)	−0.0048 (9)	−0.0011 (9)	−0.0009 (9)
C12A	0.0284 (11)	0.0287 (12)	0.0360 (12)	−0.0096 (9)	−0.0049 (9)	0.0064 (9)
C13A	0.0237 (11)	0.0456 (15)	0.0264 (11)	−0.0012 (10)	0.0042 (9)	0.0080 (10)
O4B	0.0674 (12)	0.0357 (10)	0.0378 (10)	0.0045 (8)	0.0049 (8)	0.0120 (8)
N1B	0.0414 (12)	0.0407 (12)	0.0338 (11)	0.0095 (9)	−0.0037 (9)	0.0096 (9)
C2B	0.0514 (14)	0.0278 (13)	0.0381 (13)	−0.0071 (11)	0.0001 (11)	−0.0015 (10)
C3B	0.0354 (13)	0.0572 (17)	0.0337 (13)	−0.0095 (11)	−0.0082 (10)	−0.0012 (11)
C5B	0.0343 (13)	0.0581 (17)	0.0367 (13)	−0.0181 (11)	0.0004 (10)	−0.0138 (12)
C6B	0.0626 (16)	0.0292 (13)	0.0433 (14)	−0.0040 (12)	0.0095 (12)	−0.0008 (11)

### (III) Tetrahydro-2H-1,4-oxazin-4-ium (3,5-dichlorophenoxy)acetate Geometric parameters (Å, º)

**Table:**

Cl3A—C3A	1.746 (2)	C5A—C6A	1.377 (3)
Cl5A—C5A	1.744 (2)	C12A—C13A	1.522 (3)
O11A—C1A	1.364 (3)	C2A—H2A	0.9500
O11A—C12A	1.421 (3)	C4A—H4A	0.9500
O13A—C13A	1.228 (3)	C6A—H6A	0.9500
O14A—C13A	1.257 (3)	C12A—H12A	0.9900
O4B—C6B	1.416 (3)	C12A—H13A	0.9900
O4B—C3B	1.407 (3)	C2B—C3B	1.490 (3)
N1B—C2B	1.485 (3)	C5B—C6B	1.489 (3)
N1B—C5B	1.477 (3)	C2B—H21B	0.9900
N1B—H12B	0.88 (2)	C2B—H22B	0.9900
N1B—H11B	0.88 (2)	C3B—H31B	0.9900
C1A—C2A	1.384 (3)	C3B—H32B	0.9900
C1A—C6A	1.388 (3)	C5B—H51B	0.9900
C2A—C3A	1.383 (3)	C5B—H52B	0.9900
C3A—C4A	1.370 (3)	C6B—H61B	0.9900
C4A—C5A	1.379 (3)	C6B—H62B	0.9900
			
C1A—O11A—C12A	120.24 (17)	C13A—C12A—H12A	109.00
C3B—O4B—C6B	109.96 (17)	O11A—C12A—H12A	109.00
C2B—N1B—C5B	111.72 (18)	C13A—C12A—H13A	109.00
C5B—N1B—H11B	114.7 (15)	O11A—C12A—H13A	109.00
C2B—N1B—H12B	112.3 (15)	H12A—C12A—H13A	108.00
H11B—N1B—H12B	105 (2)	N1B—C2B—C3B	110.27 (19)
C2B—N1B—H11B	106.6 (15)	O4B—C3B—C2B	111.23 (18)
C5B—N1B—H12B	106.7 (15)	N1B—C5B—C6B	109.70 (19)
O11A—C1A—C6A	114.67 (18)	O4B—C6B—C5B	111.47 (19)
C2A—C1A—C6A	120.75 (19)	N1B—C2B—H21B	110.00
O11A—C1A—C2A	124.57 (19)	N1B—C2B—H22B	110.00
C1A—C2A—C3A	117.77 (19)	C3B—C2B—H21B	110.00
Cl3A—C3A—C4A	118.09 (16)	C3B—C2B—H22B	110.00
C2A—C3A—C4A	123.29 (19)	H21B—C2B—H22B	108.00
Cl3A—C3A—C2A	118.62 (16)	O4B—C3B—H31B	109.00
C3A—C4A—C5A	117.18 (19)	O4B—C3B—H32B	109.00
Cl5A—C5A—C6A	119.03 (16)	C2B—C3B—H31B	109.00
Cl5A—C5A—C4A	118.85 (16)	C2B—C3B—H32B	109.00
C4A—C5A—C6A	122.1 (2)	H31B—C3B—H32B	108.00
C1A—C6A—C5A	118.84 (19)	N1B—C5B—H51B	110.00
O11A—C12A—C13A	113.95 (18)	N1B—C5B—H52B	110.00
O14A—C13A—C12A	115.08 (19)	C6B—C5B—H51B	110.00
O13A—C13A—O14A	125.4 (2)	C6B—C5B—H52B	110.00
O13A—C13A—C12A	119.5 (2)	H51B—C5B—H52B	108.00
C3A—C2A—H2A	121.00	O4B—C6B—H61B	109.00
C1A—C2A—H2A	121.00	O4B—C6B—H62B	109.00
C3A—C4A—H4A	121.00	C5B—C6B—H61B	109.00
C5A—C4A—H4A	121.00	C5B—C6B—H62B	109.00
C5A—C6A—H6A	121.00	H61B—C6B—H62B	108.00
C1A—C6A—H6A	121.00		
			
C12A—O11A—C1A—C2A	−8.1 (3)	C1A—C2A—C3A—Cl3A	178.48 (16)
C12A—O11A—C1A—C6A	172.70 (18)	Cl3A—C3A—C4A—C5A	−178.43 (16)
C1A—O11A—C12A—C13A	−76.5 (2)	C2A—C3A—C4A—C5A	1.5 (3)
C3B—O4B—C6B—C5B	62.3 (2)	C3A—C4A—C5A—Cl5A	−179.46 (16)
C6B—O4B—C3B—C2B	−61.6 (2)	C3A—C4A—C5A—C6A	0.2 (3)
C2B—N1B—C5B—C6B	51.4 (3)	C4A—C5A—C6A—C1A	−1.8 (3)
C5B—N1B—C2B—C3B	−51.2 (3)	Cl5A—C5A—C6A—C1A	177.83 (16)
C2A—C1A—C6A—C5A	1.8 (3)	O11A—C12A—C13A—O13A	15.0 (3)
O11A—C1A—C2A—C3A	−179.44 (19)	O11A—C12A—C13A—O14A	−167.02 (18)
C6A—C1A—C2A—C3A	−0.3 (3)	N1B—C2B—C3B—O4B	56.1 (2)
O11A—C1A—C6A—C5A	−178.92 (19)	N1B—C5B—C6B—O4B	−56.9 (2)
C1A—C2A—C3A—C4A	−1.5 (3)		

### (III) Tetrahydro-2H-1,4-oxazin-4-ium (3,5-dichlorophenoxy)acetate Hydrogen-bond geometry (Å, º)

**Table:**

*D*—H···*A*	*D*—H	H···*A*	*D*···*A*	*D*—H···*A*
N1*B*—H11*B*···O13*A*	0.88 (2)	2.07 (2)	2.892 (3)	156 (2)
N1*B*—H11*B*···O14*A*	0.88 (2)	2.26 (2)	2.988 (3)	141 (2)
N1*B*—H12*B*···O14*A*^i^	0.88 (2)	1.87 (2)	2.737 (3)	170 (2)
C12*A*—H12*A*···O13*A*^ii^	0.99	2.41	3.398 (3)	173

Symmetry codes: (i) *x*−1, *y*, *z*; (ii) *x*+1, *y*, *z*.

### (IV) Tetrahydro-2H-1,4-oxazin-4-ium (2,4-dichlorophenoxy)acetate Crystal data

**Table:**

C_4_H_10_NO^+^·C_8_H_5_Cl_2_O_3_^−^	*F*(000) = 640
*M**_r_* = 308.15	*D*_x_ = 1.456 Mg m^−^^3^
Monoclinic, *P*2_1_/*c*	Mo *K*α radiation, λ = 0.71073 Å
Hall symbol: -P 2ybc	Cell parameters from 2359 reflections
*a* = 9.3657 (5) Å	θ = 3.6–28.4°
*b* = 7.1702 (3) Å	µ = 0.47 mm^−^^1^
*c* = 21.1340 (11) Å	*T* = 200 K
β = 97.981 (5)°	Plate, colourless
*V* = 1405.48 (12) Å^3^	0.35 × 0.35 × 0.12 mm
*Z* = 4	

### (IV) Tetrahydro-2H-1,4-oxazin-4-ium (2,4-dichlorophenoxy)acetate Data collection

**Table:**

Oxford Diffraction Gemini-S CCD-detector diffractometer	2754 independent reflections
Radiation source: Enhance (Mo) X-ray source	2273 reflections with *I*.2σ(*I*)
Graphite monochromator	*R*_int_ = 0.026
Detector resolution: 16.077 pixels mm^-1^	θ_max_ = 26.0°, θ_min_ = 3.4°
ω scans	*h* = −9→11
Absorption correction: multi-scan (CrysAlis PRO; Agilent, 2014)	*k* = −8→8
*T*_min_ = 0.933, *T*_max_ = 0.980	*l* = −26→20
6400 measured reflections	

### (IV) Tetrahydro-2H-1,4-oxazin-4-ium (2,4-dichlorophenoxy)acetate Refinement

**Table:**

Refinement on *F*^2^	Primary atom site location: structure-invariant direct methods
Least-squares matrix: full	Secondary atom site location: difference Fourier map
*R*[*F*^2^ > 2σ(*F*^2^)] = 0.038	Hydrogen site location: inferred from neighbouring sites
*wR*(*F*^2^) = 0.091	H atoms treated by a mixture of independent and constrained refinement
*S* = 1.04	*w* = 1/[σ^2^(*F*_o_^2^) + (0.0405*P*)^2^ + 0.3419*P*] where *P* = (*F*_o_^2^ + 2*F*_c_^2^)/3
2754 reflections	(Δ/σ)_max_ < 0.001
178 parameters	Δρ_max_ = 0.28 e Å^−^^3^
2 restraints	Δρ_min_ = −0.26 e Å^−^^3^

### (IV) Tetrahydro-2H-1,4-oxazin-4-ium (2,4-dichlorophenoxy)acetate Special details

**Table:**

Geometry. Bond distances, angles etc. have been calculated using the rounded fractional coordinates. All su's are estimated from the variances of the (full) variance-covariance matrix. The cell esds are taken into account in the estimation of distances, angles and torsion angles
Refinement. Refinement of F^2^ against ALL reflections. The weighted R-factor wR and goodness of fit S are based on F^2^, conventional R-factors R are based on F, with F set to zero for negative F^2^. The threshold expression of F^2^ > 2sigma(F^2^) is used only for calculating R-factors(gt) etc. and is not relevant to the choice of reflections for refinement. R-factors based on F^2^ are statistically about twice as large as those based on F, and R- factors based on ALL data will be even larger.

### (IV) Tetrahydro-2H-1,4-oxazin-4-ium (2,4-dichlorophenoxy)acetate Fractional atomic coordinates and isotropic or equivalent isotropic displacement parameters (Å^2^)

**Table:**

	*x*	*y*	*z*	*U*_iso_*/*U*_eq_	
Cl2A	0.23659 (5)	0.74483 (6)	0.41042 (2)	0.0354 (2)	
Cl4A	0.15479 (6)	0.32605 (9)	0.19759 (2)	0.0500 (2)	
O11A	0.13251 (13)	0.42531 (17)	0.47171 (6)	0.0276 (4)	
O13A	0.34279 (13)	0.1659 (2)	0.50395 (7)	0.0390 (5)	
O14A	0.19747 (12)	−0.01154 (18)	0.55307 (6)	0.0313 (4)	
C1A	0.13210 (17)	0.3923 (3)	0.40846 (8)	0.0236 (5)	
C2A	0.18091 (18)	0.5368 (3)	0.37274 (9)	0.0255 (5)	
C3A	0.18796 (19)	0.5185 (3)	0.30830 (9)	0.0305 (6)	
C4A	0.14433 (19)	0.3520 (3)	0.27899 (9)	0.0319 (6)	
C5A	0.0933 (2)	0.2087 (3)	0.31247 (10)	0.0348 (6)	
C6A	0.08662 (19)	0.2287 (3)	0.37705 (9)	0.0306 (6)	
C12A	0.10285 (19)	0.2707 (3)	0.51091 (9)	0.0270 (6)	
C13A	0.22590 (18)	0.1309 (2)	0.52275 (8)	0.0226 (5)	
O4B	0.56703 (19)	0.3986 (3)	0.31170 (7)	0.0591 (6)	
N1B	0.55499 (17)	0.2047 (2)	0.42729 (8)	0.0293 (5)	
C2B	0.5720 (2)	0.4092 (3)	0.42590 (10)	0.0353 (7)	
C3B	0.6444 (3)	0.4633 (3)	0.37002 (11)	0.0509 (8)	
C5B	0.5609 (3)	0.2016 (4)	0.31253 (11)	0.0541 (9)	
C6B	0.4830 (2)	0.1326 (3)	0.36568 (11)	0.0412 (7)	
H3A	0.22200	0.61780	0.28470	0.0370*	
H5A	0.06260	0.09580	0.29130	0.0420*	
H6A	0.05060	0.12950	0.40010	0.0370*	
H12A	0.01550	0.20560	0.49020	0.0320*	
H13A	0.08180	0.31880	0.55260	0.0320*	
H11B	0.6420 (17)	0.149 (3)	0.4381 (9)	0.0350*	
H12B	0.507 (2)	0.180 (3)	0.4587 (8)	0.0350*	
H21B	0.63050	0.45170	0.46590	0.0420*	
H22B	0.47630	0.46970	0.42270	0.0420*	
H31B	0.65250	0.60090	0.36850	0.0610*	
H32B	0.74300	0.41080	0.37530	0.0610*	
H51B	0.66010	0.15050	0.31820	0.0650*	
H52B	0.51060	0.15630	0.27110	0.0650*	
H61B	0.38150	0.17540	0.35850	0.0490*	
H62B	0.48330	−0.00540	0.36630	0.0490*	

### (IV) Tetrahydro-2H-1,4-oxazin-4-ium (2,4-dichlorophenoxy)acetate Atomic displacement parameters (Å^2^)

**Table:**

	*U*^11^	*U*^22^	*U*^33^	*U*^12^	*U*^13^	*U*^23^
Cl2A	0.0474 (3)	0.0221 (3)	0.0376 (3)	−0.0043 (2)	0.0088 (2)	0.0006 (2)
Cl4A	0.0576 (4)	0.0620 (4)	0.0312 (3)	0.0069 (3)	0.0087 (2)	−0.0094 (3)
O11A	0.0339 (7)	0.0222 (7)	0.0274 (7)	0.0037 (6)	0.0072 (5)	0.0055 (6)
O13A	0.0247 (7)	0.0429 (9)	0.0524 (9)	0.0066 (6)	0.0162 (6)	0.0154 (7)
O14A	0.0291 (7)	0.0282 (7)	0.0368 (8)	0.0031 (6)	0.0056 (6)	0.0117 (6)
C1A	0.0202 (8)	0.0236 (9)	0.0265 (10)	0.0059 (7)	0.0019 (7)	0.0052 (8)
C2A	0.0228 (9)	0.0222 (9)	0.0315 (10)	0.0032 (8)	0.0036 (7)	0.0015 (8)
C3A	0.0290 (10)	0.0325 (11)	0.0307 (11)	0.0034 (9)	0.0063 (8)	0.0071 (9)
C4A	0.0295 (10)	0.0386 (12)	0.0268 (10)	0.0064 (9)	0.0010 (8)	−0.0019 (9)
C5A	0.0328 (10)	0.0302 (11)	0.0389 (12)	−0.0021 (9)	−0.0040 (9)	−0.0053 (9)
C6A	0.0283 (10)	0.0260 (10)	0.0363 (11)	−0.0026 (8)	0.0003 (8)	0.0041 (9)
C12A	0.0259 (9)	0.0278 (10)	0.0288 (10)	0.0031 (8)	0.0090 (7)	0.0080 (8)
C13A	0.0236 (9)	0.0225 (9)	0.0214 (9)	0.0011 (8)	0.0025 (7)	−0.0005 (8)
O4B	0.0774 (12)	0.0641 (12)	0.0344 (9)	−0.0058 (10)	0.0026 (8)	0.0203 (9)
N1B	0.0245 (8)	0.0311 (9)	0.0338 (9)	0.0047 (7)	0.0089 (7)	0.0114 (8)
C2B	0.0385 (11)	0.0292 (11)	0.0367 (12)	0.0044 (9)	0.0001 (9)	0.0014 (9)
C3B	0.0611 (15)	0.0443 (14)	0.0466 (14)	−0.0155 (12)	0.0046 (11)	0.0164 (12)
C5B	0.0596 (15)	0.0678 (18)	0.0337 (13)	0.0014 (14)	0.0025 (11)	−0.0126 (13)
C6B	0.0362 (11)	0.0337 (12)	0.0524 (14)	−0.0035 (10)	0.0012 (10)	−0.0015 (10)

### (IV) Tetrahydro-2H-1,4-oxazin-4-ium (2,4-dichlorophenoxy)acetate Geometric parameters (Å, º)

**Table:**

Cl2A—C2A	1.737 (2)	C5A—C6A	1.382 (3)
Cl4A—C4A	1.7466 (19)	C12A—C13A	1.522 (3)
O11A—C1A	1.357 (2)	C3A—H3A	0.9500
O11A—C12A	1.434 (2)	C5A—H5A	0.9500
O13A—C13A	1.241 (2)	C6A—H6A	0.9500
O14A—C13A	1.254 (2)	C12A—H12A	0.9900
O4B—C5B	1.414 (4)	C12A—H13A	0.9900
O4B—C3B	1.418 (3)	C2B—C3B	1.492 (3)
N1B—C2B	1.476 (3)	C5B—C6B	1.506 (3)
N1B—C6B	1.474 (3)	C2B—H21B	0.9900
N1B—H12B	0.870 (18)	C2B—H22B	0.9900
N1B—H11B	0.908 (18)	C3B—H31B	0.9900
C1A—C2A	1.396 (3)	C3B—H32B	0.9900
C1A—C6A	1.386 (3)	C5B—H51B	0.9900
C2A—C3A	1.379 (3)	C5B—H52B	0.9900
C3A—C4A	1.380 (3)	C6B—H61B	0.9900
C4A—C5A	1.371 (3)	C6B—H62B	0.9900
			
C1A—O11A—C12A	117.39 (15)	C13A—C12A—H12A	109.00
C3B—O4B—C5B	109.44 (17)	O11A—C12A—H12A	109.00
C2B—N1B—C6B	111.61 (16)	C13A—C12A—H13A	109.00
C6B—N1B—H11B	110.6 (12)	O11A—C12A—H13A	109.00
C2B—N1B—H12B	106.6 (14)	H12A—C12A—H13A	108.00
H11B—N1B—H12B	105.0 (17)	N1B—C2B—C3B	109.65 (17)
C2B—N1B—H11B	110.3 (13)	O4B—C3B—C2B	111.7 (2)
C6B—N1B—H12B	112.4 (12)	O4B—C5B—C6B	111.2 (2)
O11A—C1A—C6A	125.33 (17)	N1B—C6B—C5B	109.52 (17)
C2A—C1A—C6A	118.12 (16)	N1B—C2B—H21B	110.00
O11A—C1A—C2A	116.55 (17)	N1B—C2B—H22B	110.00
C1A—C2A—C3A	121.85 (19)	C3B—C2B—H21B	110.00
Cl2A—C2A—C3A	118.83 (16)	C3B—C2B—H22B	110.00
Cl2A—C2A—C1A	119.32 (14)	H21B—C2B—H22B	108.00
C2A—C3A—C4A	118.28 (19)	O4B—C3B—H31B	109.00
C3A—C4A—C5A	121.29 (18)	O4B—C3B—H32B	109.00
Cl4A—C4A—C3A	118.72 (15)	C2B—C3B—H31B	109.00
Cl4A—C4A—C5A	119.99 (16)	C2B—C3B—H32B	109.00
C4A—C5A—C6A	119.92 (19)	H31B—C3B—H32B	108.00
C1A—C6A—C5A	120.52 (18)	O4B—C5B—H51B	109.00
O11A—C12A—C13A	113.66 (14)	O4B—C5B—H52B	109.00
O14A—C13A—C12A	114.26 (15)	C6B—C5B—H51B	109.00
O13A—C13A—O14A	126.00 (15)	C6B—C5B—H52B	109.00
O13A—C13A—C12A	119.72 (15)	H51B—C5B—H52B	108.00
C4A—C3A—H3A	121.00	N1B—C6B—H61B	110.00
C2A—C3A—H3A	121.00	N1B—C6B—H62B	110.00
C4A—C5A—H5A	120.00	C5B—C6B—H61B	110.00
C6A—C5A—H5A	120.00	C5B—C6B—H62B	110.00
C5A—C6A—H6A	120.00	H61B—C6B—H62B	108.00
C1A—C6A—H6A	120.00		
			
C12A—O11A—C1A—C2A	−171.22 (15)	C6A—C1A—C2A—C3A	−1.6 (3)
C12A—O11A—C1A—C6A	9.2 (2)	Cl2A—C2A—C3A—C4A	179.56 (14)
C1A—O11A—C12A—C13A	72.91 (19)	C1A—C2A—C3A—C4A	0.4 (3)
C5B—O4B—C3B—C2B	−61.7 (2)	C2A—C3A—C4A—Cl4A	−179.24 (14)
C3B—O4B—C5B—C6B	61.5 (3)	C2A—C3A—C4A—C5A	0.9 (3)
C6B—N1B—C2B—C3B	−52.8 (2)	Cl4A—C4A—C5A—C6A	179.27 (15)
C2B—N1B—C6B—C5B	52.8 (2)	C3A—C4A—C5A—C6A	−0.9 (3)
O11A—C1A—C6A—C5A	−178.82 (17)	C4A—C5A—C6A—C1A	−0.5 (3)
C2A—C1A—C6A—C5A	1.6 (3)	O11A—C12A—C13A—O13A	6.5 (2)
O11A—C1A—C2A—C3A	178.80 (16)	O11A—C12A—C13A—O14A	−175.12 (14)
C6A—C1A—C2A—Cl2A	179.20 (14)	N1B—C2B—C3B—O4B	57.1 (2)
O11A—C1A—C2A—Cl2A	−0.4 (2)	O4B—C5B—C6B—N1B	−57.3 (2)

### (IV) Tetrahydro-2H-1,4-oxazin-4-ium (2,4-dichlorophenoxy)acetate Hydrogen-bond geometry (Å, º)

**Table:**

*D*—H···*A*	*D*—H	H···*A*	*D*···*A*	*D*—H···*A*
N1*B*—H11*B*···O13*A*^i^	0.91 (2)	2.56 (2)	3.115 (2)	120 (1)
N1*B*—H11*B*···O14*A*^i^	0.91 (2)	1.79 (2)	2.683 (2)	169 (2)
N1*B*—H12*B*···O13*A*	0.87 (2)	1.92 (2)	2.747 (2)	158 (2)
C12*A*—H12*A*···O14*A*^ii^	0.99	2.50	3.484 (2)	173
C2*B*—H21*B*···O11*A*^iii^	0.99	2.57	3.477 (2)	151
C5*B*—H52*B*···O4*B*^iv^	0.99	2.58	3.489 (3)	153

Symmetry codes: (i) −*x*+1, −*y*, −*z*+1; (ii) −*x*, −*y*, −*z*+1; (iii) −*x*+1, −*y*+1, −*z*+1; (iv) −*x*+1, *y*−1/2, −*z*+1/2.

## References

[bb1] Agilent (2014). *CrysAlis PRO*. Agilent Technologies Ltd, Yarnton, Oxfordshire, England.

[bb2] Altomare, A., Cascarano, G., Giacovazzo, C. & Guagliardi, A. (1993). *J. Appl. Cryst.* **26**, 343–350.

[bb3] André, V., Braga, D., Grepioni, F. & Duarte, M. T. (2009). *Cryst. Growth Des.* **9**, 5108–5116.

[bb4] Farrugia, L. J. (2012). *J. Appl. Cryst.* **45**, 849–854.

[bb5] Ishida, H., Rahman, B. & Kashino, S. (2001*a*). *Acta Cryst.* C**57**, 1450–1453. 10.1107/s0108270101016389 11740114

[bb6] Ishida, H., Rahman, B. & Kashino, S. (2001*b*). *Acta Cryst.* E**57**, o627–o629. 10.1107/s0108270101007016 11443273

[bb7] Ishida, H., Rahman, B. & Kashino, S. (2001*c*). *Acta Cryst.* E**57**, o630–o632. 10.1107/s0108270101007016 11443273

[bb8] Kennard, C. H. L., Smith, G. & White, A. H. (1982). *Acta Cryst.* B**38**, 868–875.

[bb9] Liu, H.-L., Guo, S.-H., Li, Y.-Y. & Jian, F.-F. (2009). *Acta Cryst.* E**65**, o1905. 10.1107/S1600536809026919 PMC2977423 21583595

[bb10] Lynch, D. E., Barfield, J., Frost, J., Antrobus, R. & Simmons, J. (2003). *Cryst. Eng.* **6**, 109–122.

[bb11] O’Neil, M. J. (2001). Editor. *The Merck Index*, 13th ed., pp. 1495–1496. Whitehouse Station, NJ, USA: Merck & Co. Inc.

[bb12] Sheldrick, G. M. (2008). *Acta Cryst.* A**64**, 112–122. 10.1107/S0108767307043930 18156677

[bb13] Smith, G. (2014). *Acta Cryst.* E**70**, 528–532. 10.1107/S160053681402488X PMC4257399 25552984

[bb14] Smith, G., Kennard, C. H. L. & White, A. H. (1976). *J. Chem. Soc. Perkin Trans. 2*, pp. 791–792.

[bb15] Smith, G. & Lynch, D. E. (2015*a*). *Acta Cryst.* E**71**, 671–674. 10.1107/S205698901500907X PMC4459305 26090147

[bb16] Smith, G. & Lynch, D. E. (2015*b*). Unpublished results.

[bb17] Smith, G., Lynch, D. E., Sagatys, D. S., Kennard, C. H. L. & Katekar, G. F. (1992). *Aust. J. Chem.* **45**, 1101–1108.

[bb18] Spek, A. L. (2009). *Acta Cryst.* D**65**, 148–155. 10.1107/S090744490804362X PMC2631630 19171970

[bb19] Zumdahl, R. L. (2010). In *A History of Weed Science in the United States*. New York: Elsevier.

